# Veterinary Compounding: Regulation, Challenges, and Resources

**DOI:** 10.3390/pharmaceutics9010005

**Published:** 2017-01-10

**Authors:** Gigi Davidson

**Affiliations:** Clinical Pharmacy Services, North Carolina State University, College of Veterinary Medicine, Raleigh, NC 27607, USA; gsdavid2@ncsu.edu; Tel.: +1-919-513-6575

**Keywords:** compounding, veterinary, veterinary pharmacy

## Abstract

The spectrum of therapeutic need in veterinary medicine is large, and the availability of approved drug products for all veterinary species and indications is relatively small. For this reason, extemporaneous preparation, or compounding, of drugs is commonly employed to provide veterinary medical therapies. The scope of veterinary compounding is broad and focused primarily on meeting the therapeutic needs of companion animals and not food-producing animals in order to avoid human exposure to drug residues. As beneficial as compounded medical therapies may be to animal patients, these therapies are not without risks, and serious adverse events may occur from poor quality compounds or excipients that are uniquely toxic when administered to a given species. Other challenges in extemporaneous compounding for animals include significant regulatory variation across the global veterinary community, a relative lack of validated compounding formulas for use in animals, and poor adherence by compounders to established compounding standards. The information presented in this article is intended to provide an overview of the current landscape of compounding for animals; a discussion on associated benefits, risks, and challenges; and resources to aid compounders in preparing animal compounds of the highest possible quality.

## 1. Introduction

The spectrum of therapeutic need in veterinary medicine is large, and the availability of approved drug products for all veterinary species and indications is relatively small. Consequently, compounding is of great importance to fill therapeutic gaps for non-human species. In a Guidance for Industry released in May 2015, the US Food and Drug Administration (FDA) estimated that 75,000 pharmacies fill 6,350,000 compounded prescriptions for animals in the United States each year [1]. Estimates for other countries are not available; however, a survey of veterinarians in Czechoslovakia revealed that surveyed veterinarians prescribe no more than one compounded preparation per day [2]. Although no other specific data is available, considering the many roles that animals play for humans, the prevalence of compounds prepared for animals worldwide is likely to be large.

Competence in providing pharmaceutical care and compounds for animal patients is critical for pharmacists because pharmacists are the only health care providers that are expected by society to provide care for all species—humans and non-humans, and are the only health care providers that are legally allowed to do so. Pharmacists are also well-positioned to consult with veterinarians to collaborate to provide a high quality compounded formulation that is potentially safe for the intended patient and has optimal composition to potentially result in the intended therapeutic effect. The information presented in this article is intended to provide an overview of the current landscape of compounding for animals; a discussion on associated benefits, risks, and challenges; and resources to aid in preparing animal compounds of the highest possible quality.

## 2. Definition of Compounding

Terminology to describe the extemporaneous preparation of medicines varies widely across the global community. The practice may be referred to as “extemporaneous manufacturing”, “extemporaneous preparation”, “extemporaneous compounding”, or simply “compounding” depending on the national directives of the individual countries [3,4,5]. For the purposes of this article, the term “compounding” will be used. Legal definitions for compounding are often non-specific and broad. Compounding is most commonly defined as manipulating an approved drug formulation for use in a manner that is not provided for in the drug’s approved labeling [6]. The New Zealand government defines compounding for animals as “a means to make up, prepare, produce, or process a veterinary medicine into a preparation for treatment of animals under the care of the compounding veterinarian” [7]. Compounding is usually considered when a therapy for an individual patient cannot be provided by a commercially available product. Compounding can include activities such as mixing two or more approved drug products together into a single dosage form (e.g., mixing two anesthetic drugs in the same syringe), changing the dosage form (e.g., crushing oral tablets to make an oral liquid suspension), or adding patient-preferred flavoring to an approved drug product. In the event that no approved drug exists for the desired therapy, compounding can also include starting with bulk chemical active ingredients and other excipients such as suspending agents, fillers, binders, and flavors.

## 3. Scope

Although in its proposed Guidance for Industry #230 the United States FDA estimated that 75,000 pharmacies fill 6,350,000 compounded prescriptions for animals in the US each year, the exact extent to which drugs are compounded for animals is unknown. The Brakke Company conducted a survey of veterinary compounding in 2013 and claims to answer the question: “How big is the veterinary drug compounding market?” However, results of this survey are only available by purchase for $8995.00 (USD) [8]. Factual numbers based on reporting to FDA are not available because unlike veterinary drug manufacturers, compounders preparing veterinary compounds do not have to register with FDA. While pharmacies must register with state boards of pharmacy in the United States, the data collected by state boards of pharmacy is not aggregated or comprehensive, and many boards of pharmacy do not distinguish prescription activity for humans from that activity for non-humans. While several countries require that pharmacists follow regulations or guidelines for compounding medicines and some may impose reporting requirements for the number and type of compounds prepared [9], these requirements appear to be limited to only sterile compounds prepared for humans.

## 4. Benefits of Compounding for Animals

Compounded medications provide therapy for animal patients when no suitable government-approved (e.g., The United States Food and Drug Administration) products are available. Products approved for use in certain species may be commercially available in dosage forms (e.g., large chewable tablets) that are not suitable for use in other species (e.g., cats or exotic animal patients). Likewise, approved products may be available in flavors that are not accepted by certain animal species (e.g., citrus and bubblegum flavored pediatric medicines are not accepted by cats). In these instances, compounding can be used to change an approved product into an acceptable dosage form or flavor to increase adherence in an individual patient, and particularly in species which are difficult to medicate (e.g., cats, exotic, and wild animal species).

In addition to providing medical therapy to suit the needs of an individual animal patient, compounded preparations also provide benefit when there is no approved product available (ether for humans or animals) containing the desired active ingredients. Cisapride was withdrawn from US human market for safety reasons in 2000, but is the only known safe and effective therapy to treat chronic constipation or megacolon in cats [10]. No veterinary drug companies have elected to submit applications for cisapride approval for animals, so compounding remains the only option for veterinarians to obtain cisapride for animals in need of effective prokinetic drug therapy. Drugs containing bromides were also removed from the human market in the 1970s, and potassium bromide remains unavailable in a legally marketed, approved animal dosage form. Pharmacokinetic and pharmacodynamic parameters are well-described to support the safety and efficacy of bromides for treatment of idiopathic epilepsy in dogs [11], and veterinarians commonly prescribe compounded potassium bromide therapy for their canine patients.

Economic reasons are also often cited by veterinarians as a benefit of using compounded drug therapies. Regulatory agencies and professional veterinary organizations have stated that using a compounded preparation over an approved product strictly for economic reasons is inappropriate; however, many veterinarians care for animals owned by persons who are either unable or unwilling to pay for expensive approved therapy. Very few animal patients are covered by medical insurance policies, so cost of medical care for animals is completely out of pocket for most animal owners. Veterinarians often find themselves having to choose between strict legal compliance (i.e., prescribing an expensive approved product) or relieving animal suffering and avoiding potential death by breaking the law (i.e., prescribing a compounded version of the approved product). Examples of compounded therapies that are commonly prescribed by veterinarians for economic reasons are chemotherapeutic regimens (e.g., chlorambucil for lymphoma), chronic disease (e.g., atopic dermatitis or hyperadrenocorticism), or systemic fungal infections (e.g., blastomycosis). Regardless of initial cost savings, the risk of poor quality, lack of bioavailability, and subsequent therapeutic failure from these compounded mimic therapies is significant and predictably may increase overall cost due to lack of clinical response.

## 5. Risks Associated with Compounded Therapies for Animals

While the benefits of compounded therapies for animals are well-established [12,13], risk of serious harm and therapeutic failure from compounded preparations is significant and also well-established. The deaths of 64 humans from contaminated sterile compounds in the US in 2012 [14] caused sweeping regulatory oversight for compounds prepared for humans [15]; however, little regulatory attention has been paid to minimize risks posed by compounds to animal patients. Headlines of animal deaths resulting from compounds have become more common in the world media. The deaths of 21 Polo Ponies in Florida from a ten-fold overdose of a selenium compound [16], the deaths of 4 horses and permanent injury to 6 others in Florida and Kentucky from superpotent concentrations of compounded pyrimethamine [17], and the deaths of 3 horses from a 70-fold superpotent clenbuterol compound [18] gained wide attention and prescriber concern, but little has been accomplished on the US regulatory front to reduce the risk of harm from compounded preparations for animals.

Animal suffering and death from compounded therapies may be attributed to many factors including preparation errors, contamination, chemical and physical instability, and lack of bioavailability in the target patient. Poor quality due to compounding error has been widely investigated and reported for compounds prepared for animals [19,20,21,22]. Although no distinction was made between compounds prepared for humans and animals, the Missouri Board of Pharmacy recently found that as many as one-fifth of randomly selected compounds from Missouri licensed pharmacies did not contain the amount of active ingredient (range 0%–450%) indicated on the prescription label [23]. At time of writing, no legal requirements exist in any country that require testing to demonstrate that compounded preparations meet the strength as indicated on the prescription labeling.

While extensive studies have been conducted to prove safety, efficacy, and bioavailability for drugs approved for animal use, there are no equivalent assurances for these attributes in compounded preparations. Ample evidence does exist, however, proving that many compounded preparations are not bioequivalent to approved products in animal patients and that even when administered by the same route are not bioavailable compared to approved products. Although some may ultimately achieve effective blood levels, drugs administered by the transdermal route are consistently less bioavailable than their oral equivalents [24,25,26,27,28]. Compounds prepared from active pharmaceutical ingredients (e.g., itraconazole) have also been proven to be less (or not at all) bioavailable compared to the approved products when given at the same dose by the same route [29,30]. Other studies have demonstrated a significant increase in oral bioavailability of approved drugs when compounded into different dosage forms. The bioavailability of intact mitotane tablets in Beagles was shown to increase 38 fold when crushed and suspended in an oil vehicle [31], posing a significant risk for life-threatening adrenal damage in dogs switched from tablets to compounded oral suspensions.

Humans may also face risk from compounds prepared for use in animals. Drug depletion profiles from tissues of treated food-producing animals have not been determined for compounds, and humans may be exposed to drug residues when eating these patients or their byproducts (e.g., meat, milk, eggs or honey). Veterinarians often fail to consider the risk of human exposure to compounded medications. Animals do not self-medicate. Even those dosage forms which animals self-administer, such as medicated feeds and water, are prepared by humans, so the risk of drug exposure to human caregivers is always great. Compounded formulations of drugs that were removed from the human market for safety reasons (e.g., cisapride, bromides, diethylstilbestrol, and trilostane) can cause serious adverse events in humans if exposed.

## 6. Adverse Events to Veterinary Compounds

The extent of adverse events in animals caused by compounds is unknown. There are no mandatory requirements for reporting adverse drug events in animals; any adverse event reporting is voluntary. The US FDA’s voluntary adverse event reporting form, the Veterinary Adverse Drug Reaction, Lack of Effectiveness, or Product Defect Report (Form 1932a) is a complex five page report designed for adverse event reporting for manufactured products. The 1932a form also does not include a prompt to determine if the adverse drug event was due to a compounded preparation, and the form is too sophisticated for most pet owners to be able to complete. FDA’s adverse drug event reporting system for animals is also not aggregated or comprehensive and is currently being revised. Since 2001 only 62 compound-related adverse events in animals have been reported to FDA. The wealth of information regarding reported adverse events from veterinary compounds is likely with state boards of pharmacy. However, various privacy and confidentiality laws in the states prevent boards of pharmacy from sharing this information even if they collect adverse events from compounds in animal patients.

## 7. Regulatory Oversight

Regulatory oversight for compounded veterinary therapies varies widely from country to country. The Parsemus Foundation, a small private foundation interested in compounded contraceptive therapies, recently surveyed the veterinary drug regulatory landscape of various countries. In their report surveying legal use for an compounded injectable chemical neutering agent for male dogs, Regulatory Status of Compounded Treatments, By Country [32], Parsemus characterizes countries as those “with a strong veterinary regulatory culture” (European Union, Canada, China, South Africa, Australia, and Japan), those “without a strong veterinary regulatory culture” (Nigeria, Trinidad and Tobago, Bangladesh, Fiji, Ghana, Iraq, Kenya, Nepal, Tanzania, and Sierra Leone), and those “with a special situation” regarding veterinary regulatory culture (Mexico, Bolivia, Panama, Colombia, and The United States). Parsemus states that the United States generally falls into the “with a strong veterinary regulatory culture” but that “great ambiguity exists around compounding in the U.S., with nearly all small-animal veterinarians ordering drugs compounded from bulk substances in situations that are technically contrary to FDA regulations.” Although the Parsemus survey was not validated or analyzed for statistical significance, its conclusions seem to be representative of opinions widely held by all relevant stakeholders for veterinary compounding in the US.

Specific regulations for veterinary compounding outside of the US are described for only a few countries. The Pharmacy Board of Australia has provided comprehensive guidelines for compounding of medicines [33] which includes a section on compounding veterinary medicines. The Australian guidelines instruct pharmacists to be educated in the principles of compounding for animals, and to maintain suitable information resources regarding veterinary medicine including consultations with veterinary surgeons. Australian pharmacists are also encouraged to seek legal advice to ensure that they are compounding within the parameters of the Australian AgVet Code. The Irish Pharmacy Practice Guidance Manual includes veterinary pharmacy in its guidance and requires that veterinary compounds only be prepared in response to a veterinarian’s order, and that no anticipatory compounding is allowed [34]. Denmark only allows compounding for animals pursuant to a veterinarian’s prescription and only if there is no suitable registered veterinary medical product available [35]. The Ontario College of Pharmacists publishes compounding guidelines that include some of the most specific guidance on veterinary compounding and require the same standards used when preparing compounds for humans, specific auxiliary labeling for veterinary compounds including the veterinarian’s stated withdrawal time for food-producing animals, and a prohibition of selling compounds to third parties outside of the veterinarian-client-patient relationship [36].

Although veterinarians may prepare compounds for animal patients, compounding practice is primarily performed by pharmacists. In most countries, pharmacy practice is regulated by provincial, or national boards of pharmacy and compounding activities are very well-regulated. However, in the US, pharmacies are solely regulated by state boards of pharmacy, and unless pharmacies are engaging in behavior that more closely resembles manufacturing, FDA has little jurisdiction in compounding pharmacies. Consequently, surveillance and compliance for veterinary compounding varies widely from state to state, and because pharmacies may register with multiple state boards of pharmacy and engage in interstate commerce of veterinary compounds, regulatory action has historically been extremely difficult to accomplish. The magnitude of veterinary compounding in the United States and the lack of regulatory consistency confirms the Parsemus assessment that much ambiguity exists and that veterinarians are able to order and dispense compounds that are technically at odds with FDA regulations. Compounding pharmacies may easily prepare quantities of compounds for sale to veterinary practices in which boards of pharmacy do not have jurisdiction. Veterinarians subsequently dispense these compounds as if they were approved products, and the extent of this activity drops, for the most part, beneath the regulatory radar. To further complicate matters, sweeping reform and enforcement of compounding through the US Drug Quality and Security Act of 2013 (DQSA) dramatically increased the regulatory oversight of compounding for humans, but the was written to specifically exclude regulation of compounding for animals. The resultant regulatory void for veterinary compounding has further contributed to great ambiguity in surveillance and determination of compliance in the United States.

The US Animal Medicinal Drug Use Clarification Act (AMDCA 1996) codified the extra label use of drugs, including compounds, in animal patients, but was silent on the use of bulk drug substances for compounding. Consequently, FDA promulgated rules for compounding that state that compounds prepared for animal patients must use FDA approved products as the starting ingredients, and that nothing in the regulation “shall be construed as permitting compounding from bulk drugs.” No other countries appear to have mandated a prohibition on the use of bulk drug substances for compounding, and as previously stated, have only required that veterinary compounds be prepared in the absence of a suitable approved product. From 1996 to 2015, FDA practiced regulatory discretion towards compounding with bulk drug substances through an internal compliance policy guide, CPG 608.400 [37]. However, in an attempt to harmonize enforcement with the DQSA, FDA rescinded CPG 608.400 in May 2015 and proposed new draft guidance for industry (GFI) [38] for public comment regarding use of bulk drug substances for animal compounding. At time of writing, FDA has not moved forward with guidance on compounding with bulk drug substances, and veterinary compounding enforcement in the United States continues to remain in regulatory limbo. Comparison of the CPG and the GFI indicate that FDA’s primary concerns regarding compounding with bulk drug substances are: copies of FDA approved drugs, resale of office stock compounds, and use of bulk drug substances to compound for food-producing animals. Hopefully, future guidance or a legislative initiative will provide clarity with respect to these three concerns for a country where more than 6 million compounds are prepared for animals annually.

FDA has inspected some compounding pharmacies on a “for cause” basis and has consequently issued warning letters to pharmacies found to not be in compliance. Unfortunately, a September 2015 audit by the US General Accounting Office (GAO) [39] found that FDA had not consistently documented the basis for citing these veterinary compounding infractions, and the warning letters have had little effect on improving the quality of compounds provided for veterinary patients.

Another unfortunate regulatory void in the United States is FDA’s lack of statutory authority to mandate drug recalls, including recalls for compounds found to be of unacceptable quality during inspections. Consequently, when FDA discovers compounds that may potentially cause harm, suffering, or death to animal patients, they must rely upon the willingness of the compounding pharmacy to issue a voluntary recall. FDA does provide a list of voluntarily recalled veterinary compounds at the following link: http://www.fda.gov/AnimalVeterinary/SafetyHealth/RecallsWithdrawals/default.htm.

## 8. Challenges in Veterinary Compounding

Even in an ideal regulatory environment, compounding for animals is not without challenge.

Although pharmacists are well-recognized experts in the field of human pharmacology and drug therapy, human pharmacotherapy concepts learned in pharmacy school cannot be easily extrapolated to non-human medical therapies. It has often been stated that although humans tend to anthropomorphize them, animals are not smaller, larger, furrier, feathered, scaled or finned versions of humans. In order to critically evaluate potential safety and efficacy of a compounded preparation for animals, the pharmacist must be aware of anatomical, physiological, metabolic, behavioral, genetic, dietary, and toxicological differences for the prescribed species.

Anatomical differences between species are a significant consideration for veterinary pharmacotherapy. While a comprehensive discussion of significant anatomical differences is beyond the scope of this article, those most relevant to preparing compounds are presented.

Body covering and body orientation are important anatomical considerations. Species covered with dense fur may not receive the full benefit of topical therapy, and species covered with feathers that are primarily used for insulation against heat and cold may be harmed by topical therapies that disrupt the integrity of feathers. For example, topical ointments are rarely if ever used on birds as this may lead to hyper- or hypothermia, depending on ambient temperatures. The majority of veterinary species have a horizontal body orientation instead of vertical, and the benefit of gravity does not facilitate passage of solid dosage forms to the stomach [40]. For example, large capsules or medicated treats compounded for dogs and cats may lodge on the esophageal mucosa and cause erosions prior to reaching the stomach where dilution and dissolution are accomplished. Caregivers for animals with a horizontal body orientation should be instructed to chase orally administered solids with at least 5 mL to 6 mL of liquid or with a small amount of food if compatible with the drug being administered.

Species metabolic, and consequently, toxicological differences are also important considerations for non-human compounding [40]. Glomerular filtration rate, hepatic drug-metabolizing enzymes, protein transporters, and efflux pumps in nonhuman species vary widely. An understanding of non-human drug metabolizing limitations is critical to preparing compounds for animal species.

Pharmacogenetic polymorphisms have been comprehensively described as a cause for idiosyncratic drug reactions and toxicities in humans [41,42] and much research has been dedicated to identifying these polymorphisms in animal species. Dogs have significant genetic anomalies that predispose them to toxicities, and the most well-characterized anomaly is mutation of the ABCB1-1Δ allele which affects p-glycoprotein transport mechanisms in herding breeds [43]. Breeds most likely to display this genetic anomaly include Collies, Longhaired Whippets, Australian Shepherds (standard and miniature), Shetland Sheepdogs, Old English Sheepdogs, Border Collies, Silken Windhounds, and German Shepherd Dogs [43]. Failure of the genetically altered drug efflux pump allows substrates such as ivermectin, loperamide, and chemotherapeutic drugs to cross the blood brain barrier of affected dogs causing severe central nervous system toxicity that is not observed in breeds without the genetic anomaly [44]. Domestic cats demonstrate significant inability to conjugate planar phenolic xenobiotics compared to other mammalian species, and investigations into the responsible mechanism have identified 5 gene mutations of isoform 1A6 of UDP-glucuronosyltransferase [45]. Consequently, all cats have limited ability to achieve drug transformation through conjugation with glucuronide, and substrates such as acetaminophen and phenazopyridine, ultimately are cleared through other metabolic pathways, often resulting in extremely toxic metabolites [46]. Any drug, excipient, preservative, flavor, or dye should be carefully evaluated before used in a preparation for cats. Alcohols, benzoic acid derivatives, and azo dyes are particularly problematic because they require conjugation with glucuronide for elimination. If a colored tracer is required when triturating powders to make capsules for cats, a naturally colored powder such as cyanocobalamin should be used instead of an artificially-colored dye. It is also very important to note that cats groom themselves and other cats, so these same principles should also be applied to all topical therapies intended for use on cats to prevent unintended systemic adverse effects from topically administered compounds.

Dogs are relatively deficient in the enzymes that accomplish drug acetylation, and this deficiency should be carefully evaluated prior to compounding for dogs. While the artificial sweetener, xylitol, is generally recognized as safe (GRAS) in most species, it is rapidly and completely absorbed across the gastrointestinal-blood barrier in dogs and acts like glucose, causing insulin release and a corresponding profound, often fatal, hypoglycemia. Chronic xylitol exposure can also cause severe hepatic necrosis in dogs [47]. Many drugs approved for use in humans contain xylitol in the inactive ingredients, and pharmacists may be asked to compound a copy of a commercially available drug that does not contain xylitol, such as Neurontin Oral Solution [48]. Canine mast cells are reactive to surfactants and preservatives commonly used in humans drugs (e.g., polysorbate 80 and Cremophor^®^) and can result in serious, life-threatening anaphylactic reactions in dogs [49,50]. While a comprehensive discussion of species-specific metabolic differences is beyond the scope of this article, Table 1 summarizes examples of excipients, flavors, preservatives, and dyes that should be avoided in select species and describes the resultant toxicity upon exposure.

## 9. Formula and Component Selection

While many verified and peer-reviewed compounding formulas are available for compounded dosage forms intended for human use [58], these formulas may or may not be appropriate for use in non-human species. The United States Pharmacopeia (USP) has developed dozens of verified compounded preparation formulas specifically for veterinary use [59]. USP nomenclature identifies these formulas in its compendia by the following format: Drug Generic name, Compounded, Dosage Form, Veterinary (e.g., Enrofloxacin, Compounded, Oral Suspension, Veterinary). USP veterinary compounded preparation monographs have been stability tested to the beyond-use-dates (BUDs) or discard dates published in their corresponding monographs and contain specific instructions for how to prepare, package, test, store, and label each compound. Many of the almost two hundred USP compounded preparations that are not specified for veterinary use may also be used in animal patients if the criteria presented above are used to evaluate species-specific concerns. In addition to the USP-National Formulary (USP-NF) print volumes, USP now offers an electronic USP Compounding Compendium [59] that contains all standards and formulas related to compounding.

When a USP formula monograph is not available, drug manufacturers may sometimes provide extemporaneous compounding information for their products; however, this information is rarely available, and manufacturers are often not willing to share this information due to concerns about liability. Other compounding formula evidence can be located by searching secondary source collections of published peer-reviewed compounded preparation stability studies [58], again with the caveat that species-specific considerations should be applied to each component utilized in a published compounded preparation formula. If peer-reviewed evidence cannot be located in secondary resources, a search of primary peer-reviewed literature may reveal a stability-tested formula that is suitable for use in animal patients. The International Journal of Pharmaceutical Compounding (Loyd V. Allen, Editor-in-Chief, Edmond, OK, USA) is a bimonthly scientific and professional journal that frequently features stability-tested formulas for veterinary compounds.

When no evidence is available to support stability and ingredient compatibility for a compounded preparation, USP or other compendial compounding defaults should be applied after careful consideration of the inherent stability of the active drug, suitability of components for the target patient/species, and concerns for adverse effects if the compound is not stable throughout the labeled beyond use date (BUD) and intended therapy period.

## 10. Conclusions

Compounding is a critical component of providing veterinary medical therapy. The global animal pharmaceutical industry and approval agencies can never be expected to keep up with the number of species and indications for which medical therapies are required. Consequently, compounding will continue to bridge therapeutic gaps in veterinary medicine. Because federal regulation of veterinary medicine is largely focused on avoidance of drug residues in food-producing animals, use of compounds in companion animals is not closely monitored. And because compounds for companion animals are almost exclusively prepared by pharmacists, state and provincial boards of pharmacy have the primary responsibility for regulating this practice, but little ability to do so as most of these compounds end up in veterinary practices as office stock. Regulatory gaps between veterinary medicine and pharmacy will be difficult to bridge in most municipalities. Until such surveillance is in place, pharmacists must self-educate to become aware of the unique species idiosyncrasies of non-human species and use all available resources to provide safe, effective, and high quality compounded preparations.

## Figures and Tables

**Table 1:** Toxic drugs, excipients and foods by species and resultant toxicity.

Drug/Excipient/Food	Species Affected	Toxicity
Avocado [51]	Birds	Pulmonary congestion, non-suppurative inflammation of the liver, kidney, pancreas, skin, and proventriculus
Benzocaine, benzoic acid derivatives [52]	Cats	Red blood cell oxidative injury, hemolytic anemia
Chocolate [53]	Dogs, birds	Cardiovascular and central nervous system stimulation (artificial flavors are not toxic but encourage an attraction to the natural substance)
Cremophor [49]	Dogs	Histamine release, anaphylaxis
Garlic, onions [54]	Dogs, cats	Hemolytic anemia (artificial flavors are not toxic but encourage an attraction to the natural substance)
Grapes, raisins [55]	Dogs	Renal toxicity (artificial flavors are not toxic but encourage an attraction to the natural substance)
Macadamia nuts [56]	Dogs	Lethargy, hyperthermia, ataxia, vomiting (artificial flavors are not toxic but encourage an attraction to the natural substance)
Polysorbate 80 [50]	Dogs	Histamine release, anaphylaxis
Xylitol [57]	Dogs, birds	Profound hypoglycemia and hepatocellular necrosis

## References

[1] Compounding Animal Drugs From Bulk Drug Substances; Draft Guidance for Industry; Availability; Withdrawal of Compliance Policy Guide; Section 608.400 Compounding of Drugs for Use in Animals. https://www.federalregister.gov/articles/2015/05/19/2015-11982/compounding-animal-drugs-from-bulk-drug-substances-draft-guidance-for-industry-availability.

[2] Agelova J., Maceskova B. (2005). Analysis of drugs used in out-patient practice of veterinary medicine. Ceska Slova Farm..

[3] World Health Organization WHO Expert Committees 18^th^ Expert Committee on the Selection and Use of Essential Medicines. Extemporaneous Review. http://www.who.int/selection_medicines/committees/expert/18/policy/policy5/en/.

[4] European Directive 65/65/CE. http://eur-lex.europa.eu/LexUriServ/LexUriServ.do?uri=CELEX:31965L0065:EN:HTML.

[5] The US Food and Drug Administration What is compounding?. http://www.fda.gov/Drugs/GuidanceComplianceRegulatoryInformation/PharmacyCompounding/ucm339764.htm#what.

[6] Allen L.V. (2003). A history of pharmaceutical compounding. Secundum. Artem..

[7] Developing a documented system for compounded veterinary preparations. http://www.foodsafety.govt.nz/elibrary/industry/Compounding-Veterinary-Preparation.pdf.

[8] Veterinary Drug Compounding. http://www.brakkeconsulting.com/news_article/771.aspx.

[9] The US Food and Drug Administration Outsourcing Facilities. http://www.fda.gov/Drugs/GuidanceComplianceRegulatoryInformation/PharmacyCompounding/ucm393571.htm.

[10] Washabau R., Holt D. (1999). Pathogenesis, diagnosis, and therapy of feline idiopathic megacolon. Vet. Clin. N. A. Small Anim. Pract..

[11] Trepanier L.A., Van Schoick A., Schwark W.S. (1998). Therapeutic serum drug concentrations in epileptic dogs treated with potassium bromide alone or in combination with other anticonvulsants: 122 cases (1992–1996). J. Am. Vet. Med. Assoc..

[12] Frank H. (2006). Compounding in the exotic practice. J. Exot. Pet Med..

[13] Papich M.G. (2005). Drug compounding for veterinary patients. AAPS J..

[14] Centers for Disease Control and Prevention (CDC) (2012). Multistate outbreak of fungal infection associated with injection of methylprednisolone acetate solution from a single compounding pharmacy-United States, 2012. MMWR Morb. Mortal. Wkly. Rep..

[15] (2013). Drug Quality and Security Act, US Public Law 113-54.

[16] Belainesh D., Maldonado G., Reid H. (2011). Acute selenium toxicosis in polo ponies. J. Vet. Diagn. Investig..

[17] Four Horses die after Receiving Compounded EPM Drug. http://www.veterinarypracticenews.com/May-2014/4-Horses-Die-After-Receiving-Compounded-EPM-Drug/.

[18] Thompson J.A., Mirza M.H., Barker S.A., Morgan T.W., Bauer R.W., McConnico R.S. (2011). Clenbuterol Toxicosis in Three quarter horse racehorses after administration of a compounded product. J. Am. Vet. Med. Assoc..

[19] Cook A.K., Nieuwoudt C.D., Longhofer S.L. (2012). Pharmaceutical evaluation of compounded trilostane products. J. Am. Anim. Hosp. Assoc..

[20] Umstead M.E., Boothe D.M., Cruz-Espindola C., Macdonald J.M., Kennis R., Angarano D. (2012). Accuracy and precision of compounded ciclosporin capsules and solution. Vet. Dermatol..

[21] Scott-Moncrief J.C., Moore G.E., Coe J., Lynn R.C., Gwin W., Petzold R. (2012). Characteristics of commercially manufactured and compounded protamine zinc insulin. J. Am. Vet. Med. Assoc..

[22] Stanley S.D., Thomas S.M., Skinner W. Comparison for Pharmaceutical Equivalence of FDA-Approved Products and Compounded Preparations of Ketoprofen, Amikacin, and Boldenone. Proceedings of the 49th Annual Convention of the American Association of Equine Practitioners.

[23] Missouri Board of Pharmacy, Annual Reports from 2006–2014. http://pr.mo.gov/pharmacists-annual-reports.asp.

[24] Bennett N., Papich M.G., Hoenig M., Fettman M.J., Lappin M.R. (2005). Evaluation of transdermal application of glipizide in a pluronic lecithin gel to healthy cats. Am. J. Vet. Res..

[25] Ciribassi J., Luescher A., Pasloske K.S., Robertson-Plouch C., Zimmerman A., Kaloostian-Whittymore L. (2003). Comparative bioavailability of fluoxetine after transdermal and oral administration to healthy cats. Am. J. Vet. Res..

[26] Krotscheck U., Boothe D.M., Boothe H. (2004). Evaluation of transdermal morphine and fentanyl pluronic lecithin organogel administration in dogs. Vet. Ther..

[27] MacGregor J.M., Rush J.E., Rozanski E.A., Boothe D.M., Belmonte A.A., Freeman L.M. (2008). Comparison of pharmacodynamic variables following oral versus transdermal administration of atenolol to healthy cats. Am. J. Vet. Res..

[28] Mealey K.L., Peck K.E., Bennett B.S., Sellon R.K., Swinney G.R., Melzer K., Gokhale S.A., Krone T.M. (2004). Systemic absorption of amitriptyline and buspirone after oral and transdermal administration to healthy cats. J. Vet. Intern. Med..

[29] Mawby D.I., Whittemore J.C., Genger S., Papich M.G. (2014). Bioequivalence of orally administered generic, compounded, and innovator-formulated itraconazole in healthy dogs. J. Vet. Intern. Med..

[30] Smith J.A., Papich M.G., Russell G., Mitchell M.A. (2010). Effects of compounding on pharmacokinetics of itraconazole in blackfooted penguins (spheniscus demersus). J. Zoo Wildl. Med..

[31] Watson A.D.J., Rijnberk A., Moolenaar A.J. (1987). Systemic availability of o’-DDD in normal dogs, fasted and fed, and in dogs with hyperadrenocorticism. Res. Vet. Sci..

[32] The Parsemus Foundation Regulatory Status of Compounded Treatments, By Country. https://www.parsemusfoundation.org/wp-content/uploads/2015/03/CaCl2_Regulatory_Status_Opinion_10-14-2016.pdf.

[33] Guidelines on compounding of medicines. https://www.google.com/search?q=australian+guidelines+on+compounding+medicines&oq=australian+guidelines+on+compounding+medicines&aqs=chrome..69i57.13710j0j8&sourceid=chrome&ie=UTF-8#.

[34] Pharmacy Practice Guidance Manual. Veterinary Pharmacy. http://www.thepsi.ie/libraries/publications/pharmacy_practice_guidance_manual.sflb.ashx.

[35] Distribution and use of Veterinary Drugs in Denmark. https://www.foedevarestyrelsen.dk/english/Animal/AnimalHealth/Veterinary_medicine/Pages/default.aspx.

[36] Ontario College of Pharmacists Guidelines for Compounding Preparations. https://www.google.com/search?q=guidelines+for+patient+compounding+ontario&oq=guidelines+for+patient+compounding+ontario&aqs=chrome..69i57.6263j0j8&sourceid=chrome&ie=UTF-8#.

[37] FDA Manual of Compliance Policy Guides. http://www.fda.gov/ICECI/ComplianceManuals/CompliancePolicyGuidanceManual/.

[38] FDA Draft Guidance for Industry, #230. http://www.fda.gov/downloads/animalveterinary/guidancecomplianceenforcement/guidanceforindustry/ucm446862.pdf.

[39] Government Accounting Office: Drug Compounding for Animals. http://www.gao.gov/assets/680/672748.pdf.

[40] Baggot J.D. (2001). The Physiological Basis of Veterinary Clinical Pharmacology.

[41] Meyer U.A., Zanger U.M. (1997). Molecular mechanisms of genetic polymorphisms of drug metabolism. Ann. Rev. Pharmacol. Toxicol..

[42] Spielberg S.P. (1984). In vitro assessment of pharmacogenetic susceptibility to toxic drug metabolites in humans. Fed Proc..

[43] Mealey K.L., Meurs K.M. (2008). Breed distribution of the ABCB1-1Δ (multidrug sensitivity) polymorphism among dogs undergoing ABCB1 genotyping. J. Am. Vet. Med. Assoc..

[44] Washington State University College of Veterinary Medicine Veterinary Clinical Pharmacology Lab Problem Drugs. https://vcpl.vetmed.wsu.edu/problem-drugs.

[45] Greenblatt D.J. (2000). Molecular genetic basis for deficient acetaminophen glucuronidation by cats: UGT1A6 is a pseudogene, and evidence for reduced diversity of expressed hepatic UGT1A isoforms. Pharmacogenet. Genom..

[46] Walton R.P., Lawson E.H. (1934). Pharmacology and toxicology of the azo dye, phenyl-azo-alpha-alpha-diaminopyridine (Pyridium). J. Pharmacol. Exp. Ther..

[47] Piscitelli C.M., Dunayer E.K., Aumann M. (2010). Xylitol toxicity in dogs. Compend. Contin. Educ. Vet..

[48] (2009). Neurontin Oral Solution [package insert].

[49] Lorenz W., Reimann H.J., Schmal A., Dormann P., Schwarz B., Neugebauer E., Doenicke A. (1977). Histamine release in dogs by Cremophor E1 and its derivatives: Oxethylated oleic acid is the most effective constituent. Agents Actions..

[50] Varma R.K., Kaushal R., Junnarkar A.Y., Thomas G.P., Naidu M.U., Singh P.P., Tripathi R.M., Shridhar D.R. (1985). Polysorbate 80: A pharmacological study. Arzneimittelforschung.

[51] Hargis A.M., Stauber E., Casteel S., Eitner D. (1989). Avocado (Persea americana) intoxication in caged birds. J. Am. Vet. Med. Assoc..

[52] Bedford P.G.C., Clarke E.G.C. (1972). Experimental benzoic acid poisoning in the cat. Vet. Record.

[53] Gwaltney-Brant S. (2001). Chocolate intoxication. Vet. Med..

[54] Munday R., Manns E. (1994). Comparative toxicity of prop(en)yl disulfides derived from Alliaceae: Possible involvement of 1-propenyl disulfides in onion-induced hemolytic anemia. J. Agric. Food Chem..

[55] Eubig P.A., Brady M.S., Gwaltney-Brant S.M., Khan S.A., Mazzaferro E.M., Morrow C.M. (2005). Acute renal failure in dogs after the ingestion of grapes or raisins: A retrospective evaluation of 43 dogs (1992–2002). J. Vet. Intern. Med..

[56] McKenzie R.A., Purvis-Smith G.R., Allan S.J., Czerwonka-Ledez B.J., Hick L.M., Dunn M.S., Day C.T. (2000). Macadamia nut poisoning of dogs. Aust. Vet. Pract..

[57] Todd J.M., Powell L.L. (2007). Xylitol intoxication associated with fulminant hepatic failure in a dog. J. Vet. Emerg. Crit. Care.

[58] Trissel L.A. (2012). Trissel’s Stability of Compounded Formulations.

[59] United States Pharmacopeia Compounding Compendium United States Pharmacopeial Convention website. http://www.usp.org/store/products-services/usp-compounding-compendium.

